# The role of medication adherence in the association between depressive symptoms and quality of life in older adults with type 2 diabetes mellitus

**DOI:** 10.1186/s12877-023-03929-8

**Published:** 2023-03-30

**Authors:** Hao Yang, Fangtuan Wu, Mingdong Gui, Yuwei Cheng, Li Zhang

**Affiliations:** Department of Geriatric Endocrinology, The First Affiliated Hospital of Anhui Medical University, No.218 of Jixi Road, Shushan District, Hefei, 230032 Anhui China

**Keywords:** Type 2 diabetes mellitus, Quality of life, Depressive symptoms, Medication adherence

## Abstract

**Background:**

At present, the role of medication adherence in the association between depressive symptoms and quality of life (QOL) in older adults with type 2 diabetes mellitus (T2DM) was unclear. The purpose of this study was to explore the associations among depressive symptoms, medication adherence and QOL in older adults with T2DM.

**Methods:**

In this cross-sectional study, 300 older adults with T2DM from the First Affiliated Hospital of Anhui Medical University were enrolled. Among them, 115 patients had depressive symptoms and 185 had no depressive symptoms. Univariate linear regression analysis was conducted to identify possible covariates. Univariate and multivariable linear regression analyses were performed to explore the associations between depressive symptoms and medication adherence or QOL in older adults with T2DM. Multiplicative interaction analysis was evaluated whether there was interaction effect between medication adherence and depressive symptoms on QOL of patients. Mediating effect analysis was used to analyze the medication effect of medication adherence on depressive symptoms and QOL in older adults with T2DM.

**Results:**

Decreased medication adherence was observed in patients with depressive symptoms (β = -0.67, 95%CI: -1.10, -0.24) after adjusting for covariates. Depressive symptoms were associated with decreased QOL in older adults with T2DM (β = -5.99, 95%CI: -7.56, -4.42). The mediating analysis revealed that depressive symptoms were associated with decreased medication adherence (β = -0.67, 95%CI: -1.09, -0.25). Medication adherence was linked with increased QOL of older adults with T2DM (β = 0.65, 95%CI: 0.24, 1.06). Depressive symptoms were correlated with decreased QOL of older adults with T2DM (β = -5.56, 95%CI: -7.10, -4.01). The percentage mediated by medication adherence on depressive symptoms and QOL in older adults with T2DM was 10.61%.

**Conclusion:**

Medication adherence might mediate depressive symptoms and QOL of older adults with T2DM, which might provide a reference for the improvement of QOL of these patients.

**Supplementary Information:**

The online version contains supplementary material available at 10.1186/s12877-023-03929-8.

## Background

Diabetes mellitus (DM) is a chronic metabolic disease characterized by hyperglycemia and caused by various reasons [1]. Type 2 diabetes mellitus (T2DM) is the most prevalent type of DM, accounting for 90%-95% of all DM cases [2]. T2DM is epidemic globally with an increasing prevalence over the past decades [3]. A previous study estimated that there might be 592 million T2DM patients all over the world by the year of 2035 [4]. T2DM causes a substantial burden and economic losses to patients and society, making it one of the most serious public health problems in the world. T2DM is a common chronic disease in older adults, accompanied with many complications, such as diabetic neuropathy, nephropathy, retinopathy or vascular disease, which greatly affects the physical and mental health of elderly patients, and further decreases their quality of life (QOL) [5]. T2DM patients were reported to have worse QOL compared with the general population [6]. A prospective cohort study revealed that poor QOL was strongly associated with higher mortality in elderly T2DM patients [7]. To deeply uncover more modifiable factors associated with QOL improvement in older patients with T2DM is of great importance.

Depressive symptoms are a major comorbidity of T2DM, and T2DM patients had higher risk of having depressive symptoms [8]. T2DM patients with depression were found to have higher levels of hyperglycemia and cognitive impairment than T2DM patients [9]. Depressive symptoms in T2DM patients were associated with increased complications, and lowered the QOL of patients [10]. Gonzalez et al. revealed that patients with anxious depression tended to have reduced medication adherence, and anxiety was associated with poor glycemic control in patients with T2DM in Mexican population [11]. Medication adherence is an important modifiable factor affecting the control effect of T2DM, and studies unveiled that medication adherence was not only correlated with better blood sugar control [12], but also had a positive correlation with QOL in patients [13]. These data suggested that depressive symptoms and medication adherence might be important factors associated with QOL of T2DM patients. At present, whether there were associations among depressive symptoms, medication adherence and QOL in older adults with T2DM was still unclear. In addition, current studies only evaluated the associations of depressive symptoms with overall medication adherence and overall QOL of patients, the associations of depressive symptoms with detailed aspects of medication adherence and QOL of older adults with T2DM need exploration. In the present study, the role of medication adherence in the association between depressive symptoms and QOL in older adults with T2DM were analyzed based on the data of T2DM patients in our hospital. We speculated that medication adherence might have an interaction with depressive symptoms, and the interaction might be associated with the QOL in older adults with T2DM.

There was other evidence indicated that depression patients with low medication adherence are at risk for treatment failure and poor QOL [14]. A previous study reported that in people with diabetes, depressive symptoms and non-adherence were associated at higher levels of obstructive family behaviors [15]. Mazzeschi et al. found that adherence to a multidisciplinary lifestyle intervention in obese people with depressive symptoms was associated with beneficial effects on several mental domains of health-related QOL [16]. Therefore, we also speculated that there might also be an elevated risk of lower QOL in T2DM patients with depressive symptoms and non-adherence to medication.

In this study, the medication adherence was determined according to the Morisky Medication Adherence Scale-8 (MMAS-8) [17] and World Health Organization Quality of Life questionnaire (WHOQOL-BREF) [18] was applied for measuring the QOL of older adult patients with T2DM. The total score and the score of the subscales were evaluated as different outcomes. As different items of MMAS-8 and WHOQOL-BREF referred to different aspects of medication adherence and QOL of people, respectively. To deep understating the association between medication adherence and depressive symptoms on different aspects of QOL in older adults with T2DM, the total score and the score of the subscales were evaluated as different outcomes.

## Methods

### Study design and population

This cross-sectional study collected the data of 300 patients from Department of Geriatric Endocrinology, the First Affiliated Hospital of Anhui Medical University between April 2020 to November 2021. The inclusion criteria were patients with age ≥ 60 years old who meet the World Health Organization (WHO) diagnostic criteria for T2DM with the course of T2DM ≥ 1 year. Patients who suffered from serious diseases such as cancer, heart disease, liver disease and kidney failure that may affect their QOL, and those who were diagnosed with dementia or schizophrenia were not included. In total, 300 participants fulfilling the inclusion criteria were recruited for the study. This study got the approval from the Ethics Committee of the First Affiliated Hospital of Anhui Medical University (Approval No: Quick-PJ 2022–11-24). Written informed consent of the patients was obtained.

### Potential covariates

According to the findings in previous relevant studies [19–21], variables might related to the QOL in older adults with T2DM were considered as potential covariates, which included gender, age (years), height (m), weight (kg), body mass index (BMI, kg/m^2^), marital status (married, divorced or widowed), education (high school or above, or under the high school), living alone or not, smoking (never, smoking at present or smoking before), drinking (never, drinking at present or drinking before), duration of T2DM (year), complicated with hypertension, dyslipidemia or other diseases such as coronary heart disease or arrhythmology (yes or no), first-degree family history of diabetes (yes or no), diabetic retinopathy (yes or no), diabetic peripheral neuropathy (yes or no), diabetic peripheral vasculopathy (yes or no), diabetic ketoacidosis (yes or no), diabetic nephropathy (yes or no), treatments for T2DM (non-drug therapy: diet, and exercise therapy etc., oral hypoglycemic drugs combined with insulin therapy, oral hypoglycemic drugs, or insulin therapy), fasting plasma glucose (FPG, mmol/L), 2-h postprandial glucose (2hPG, mmol/L), glycated hemoglobin (HbA1c, %), total cholesterol (TC, mmol/L), triglycerides (TG, mmol/L), low density lipoprotein (LDL, mmol/L) and high density lipoprotein (HDL, mmol/L).

### Main variables and outcome variable

Depressive symptoms and medication adherence were main variables and QOL of patients was outcome in the current study.

Depressive symptoms were evaluated at admission based on Hamilton Depression Scale-17 (HAMD-17) [22]. HAMD-17 had a score of 0–52 points and higher score indicated more severe depressive symptoms [23]. When the enrolled patients admitted to our hospital, clinicians asked them the questions in the HAMD-17, and evaluated the depressive symptoms status of patients based on the answers of patients. The scale was to evaluate the depressive symptoms status of patients during the two weeks before admission to our hospital. According to the HAMD-17, a score of < 8 indicated no depressive symptoms, a score of 8 to 17 indicated mild depressive symptoms, a score of 18 to 24 indicated moderate depressive symptoms, and a score ≥ 25 indicated severe depressive symptoms. In our study, patients were divided into non-depressive symptoms group (a score of < 8) and depressive symptoms group (a score of ≥ 8).

Medication adherence was determined according to the MMAS-8 [17]. There were 8 items in the scale, which concluded that people who were failure to adhere to a medication regimen might because of several factors such as “do you sometimes have problems remembering to take your medication?”do you sometimes forget to take your medication?” and problems with the complexity of the medical regimen such as, “do you ever feel hassled about sticking to your treatment plan?” The questions are phrased to avoid the “yes‐saying” bias by reversing the wording of the questions about the way patients might experience failure in following their medication regimen, since there is a tendency for patients to give their physicians or other health care providers positive answers. Each item measures a specific medication‐taking behavior and not a determinant of adherence behavior. Response categories are yes/no for each item with a dichotomous response and a 5‐point Likert response for the last item [24]. The alternative answers to items 1 to 7 (M1-M7) were “yes/no”, and all of them were scored in reverse except for item 5 (M5), that is, the answer to “yes” in item 5 (M5) was scored 1 point, and the answer to “no” was scored 0 point. The answer to “yes” in item 1 to 7 (M1-M7) was scored 0 point and the answer to “no” was scored 1 point. Item 8 (M8) is a 5-point Likert-scale rating. “never” is worth 1 point, “almost never” 0.75 point, “sometimes” 0.5 point, “often” 0.25 point, and “always” 0 point. The total score ranges from 0 to 8, with higher scores indicating better adherence (Supplementary Table 1). Herein, when the enrolled patients admitted to hospital, clinicians asked them the questions in the MMAS-8, and evaluated the medication adherence status of patients based on the answers of patients. The scale was to evaluate the adherence status of patients during the two weeks before admission to our hospital. MMAS-8 values of < 6 were classified as “low adherence”, 6–8 as “moderate to high adherence”.

WHOQOL-BREF was applied for measuring the QOL of older adult patients with T2DM [18]. Items 1 and 2 referred to individuals’ overall perceptions of QOL and their own health; the remaining entries were divided into 4 domains including physical, psychological, social, and environmental. Physical domains: item 3, 4, 10, 15, 16, 17, 18, psychological domains: item 5, 6, 7, 11, 19, 26, social domains: item 20, 21, 22, and environmental domains: item 8, 9, 12, 13, 14, 23, 24, 25. The answers to each item were divided into 5 levels, ranging from 1 to 5, and the scores of each dimension were obtained by multiplying the average score of the item by 4. In the present study, QOL was divided into QOL 1 (item 1, Q1), QOL 2 (item 2, Q2), QOL 3 (physical domains, Q3), QOL 4 (psychological domains, Q4), QOL5 (social domains, Q5), and QOL 6 (environmental domains, Q6) (Supplementary Table 2). In the present study, when the enrolled patients admitted to hospital, clinicians asked them the questions in the WHOQOL-BREF, and evaluated the QOL of patients based on the answers of patients. The scale assessed the QOL of patients during the two weeks before admission to our hospital.

Cronbach’s α was calculated to evaluate the internal consistency of MMAS-8 and WHOQOL-BREF. A general accepted rule is that α of 0.6–0.7 suggests an acceptable level of reliability [25]. In this study, the α of MMAS-8 was 0.68, which was higher than the scale excluding each item (Supplementary Table 3). The α of WHOQOL-BREF was 0.79, which was also higher than the scale excluding each item (Supplementary Table 3). These findings suggested the good acceptable level of reliability of the two scales.

### Statistical analysis

Mean ± standard deviation (SD) was applied for displaying continuous data with normal distribution, and t-test was used for comparisons between groups. Non-normally distributed continuous data were expressed as M (Q_1_, Q_3_), and differences were compared via Wilcoxon rank sum test. Categorical data were shown as n (%) and chi-square test or Fisher’s exact probability method were utilized for difference comparisons between groups. Missing values were shown in Supplementary Table 4 and manipulated via multiple interpolation, and sensitivity analysis was performed to compare the data before and after multiple interpolation. Univariate linear regression was conducted to identify possible covariates. Univariate and multivariable linear analyses were performed to explore the associations between depressive symptoms and medication adherence or QOL in older adults with T2DM. Multiplicative interaction analysis was evaluated whether there was interaction effect between medication adherence and depressive symptoms on QOL of patients. Mediating effect analysis via stepwise testing of regression coefficients was used to analyze the medication effect of medication adherence on depressive symptoms and QOL in older adults with T2DM. [Step 1: Evaluate the total effect of independent variable X on dependent variable Y; Step 2: Evaluate the relationship between independent variable X and intermediary variable M; Step 3: After controlling the intermediary variable M, evaluate the effect of independent variable X on dependent variable Y and the effect of M on Y; The mediating effect was confirmed by: 1. The total effect is significant; 2. The effect of independent variable X on intermediary variable M is significant; 3. If the above two are satisfied, the mediating effect is significant; 4. When 1 and 2 are satisfied and the mediating variable M is controlled, the effect of independent variable X on dependent variable Y is not significant, which is called complete mediating effect [26]. β [95% confidence interval (CI)] and percentage mediated were applied to evaluate the results. R version 4.0.3 (2020–10-10) was employed for interpolation of data (MICE package). SAS 9.4 M5 (SAS Inc.) was used for data analysis.

## Results

### Comparisons of characteristics of T2DM patients with and without depressive symptoms

In total, 300 older adults with T2DM were involved in the present study, and among them, 115 patients had depressive symptoms and 185 had no depressive symptoms. The mean weight (65.25 kg vs 68.40 kg), BMI (23.63 kg/m^2^ vs 24.76 kg/m^2^), 2hPG (15.97 mmol/L vs 17.49 mmol/L) in the depressive symptoms group were lower than the non-depressive symptoms group. The median LDL in the depressive symptoms group was lower than the non-depressive symptoms group. The mean QOL (50.65 vs 56.78) and MMAS (5.72 vs 6.30) in the depressive symptoms group were lower than the non-depressive symptoms group (Table 1). The mean age of participants with MMAS-8 < 6 was lower than MMAS-8 ≥ 6 group (69.39 years vs 71.35 years). The mean height in the MMAS-8 < 6 group was higher than MMAS-8 ≥ 6 group (1.68 m vs 1.65 m). The mean QOL in the MMAS-8 < 6 group was lower than MMAS-8 ≥ 6 group (52.47 vs 55.63) (Supplementary Table 5).

**Table 1:** Comparisons of characteristics of T2DM patients with and without depressive symptoms

Variables	Total (*n* = 300)	Non-depressive symptoms (*n* = 185)	Depressive symptoms (*n* = 115)	Statistics	*P*
Gender, n (%)				χ^2^ = 1.681	0.195
Male	186 (62.00)	120 (64.86)	66 (57.39)		
Female	114 (38.00)	65 (35.14)	49 (42.61)		
Age (years), Mean ± SD	70.60 ± 8.25	70.43 ± 8.10	70.88 ± 8.53	t = -0.45	0.650
Height (m), Mean ± SD	1.66 ± 0.08	1.66 ± 0.08	1.66 ± 0.08	t = -0.09	0.926
Weight (kg), Mean ± SD	67.19 ± 10.57	68.40 ± 10.63	65.25 ± 10.24	t = 2.53	0.012
BMI (kg/m^2^), Mean ± SD	24.32 ± 3.05	24.76 ± 2.96	23.62 ± 3.07	t = 3.20	0.002
Marital status, n (%)				-	0.724
Divorced	5 (1.67)	3 (1.62)	2 (1.74)		
Widowed	32 (10.67)	22 (11.89)	10 (8.70)		
Married	263 (87.67)	160 (86.49)	103 (89.57)		
Education, n (%)				χ^2^ = 0.528	0.467
High school or above	198 (66.00)	125 (67.57)	73 (63.48)		
Under the high school	102 (34.00)	60 (32.43)	42 (36.52)		
Living alone, n (%)				χ^2^ = 1.568	0.211
Yes	26 (8.67)	19 (10.27)	7 (6.09)		
No	274 (91.33)	166 (89.73)	108 (93.91)		
Smoking, n (%)				χ^2^ = 1.251	0.535
Never	180 (60.00)	107 (57.84)	73 (63.48)		
Smoking at present	47 (15.67)	32 (17.30)	15 (13.04)		
Smoking before	73 (24.33)	46 (24.86)	27 (23.48)		
Drinking, n (%)				χ^2^ = 4.656	0.097
Never	162 (54.00)	91 (49.19)	71 (61.74)		
Drinking at present	60 (20.00)	42 (22.70)	18 (15.65)		
Drinking before	78 (26.00)	52 (28.11)	26 (22.61)		
Duration of T2DM (year), M (Q_1_, Q_3_)	15.00 (8.50, 20.00)	14.00 (8.00, 20.00)	16.00 (10.00, 20.00)	Z = 1.522	0.128
Hypertension, n (%)				χ^2^ = 0.463	0.496
No	85 (28.33)	55 (29.73)	30 (26.09)		
Yes	215 (71.67)	130 (70.27)	85 (73.91)		
Dyslipidemia, n (%)				χ^2^ = 1.402	0.236
No	125 (41.67)	82 (44.32)	43 (37.39)		
Yes	175 (58.33)	103 (55.68)	72 (62.61)		
Other diseases, n (%)				χ^2^ = 0.393	0.531
No	44 (14.67)	29 (15.68)	15 (13.04)		
Yes	256 (85.33)	156 (84.32)	100 (86.96)		
First-degree family history of diabetes, n (%)				χ^2^ = 0.068	0.794
No	175 (58.33)	109 (58.92)	66 (57.39)		
Yes	125 (41.67)	76 (41.08)	49 (42.61)		
Diabetic retinopathy, n (%)				χ^2^ = 1.209	0.272
No	273 (91.00)	171 (92.43)	102 (88.70)		
Yes	27 (9.00)	14 (7.57)	13 (11.30)		
Diabetic peripheral neuropathy, n (%)				χ^2^ = 1.785	0.182
No	108 (36.00)	72 (38.92)	36 (31.30)		
Yes	192 (64.00)	113 (61.08)	79 (68.70)		
Diabetic peripheral vasculopathy, n (%)				-	0.288
No	3 (1.00)	3 (1.62)	0 (0.00)		
Yes	297 (99.00)	182 (98.38)	115 (100.00)		
Diabetic ketoacidosis, n (%)				-	1.000
No	298 (99.33)	184 (99.46)	114 (99.13)		
Yes	2 (0.67)	1 (0.54)	1 (0.87)		
Diabetic nephropathy, n (%)				χ^2^ = 0.552	0.458
No	175 (58.33)	111 (60.00)	64 (55.65)		
Yes	125 (41.67)	74 (40.00)	51 (44.35)		
Treatments for T2DM, n (%)				-	0.097
Non-drug therapy	8 (2.67)	4 (2.16)	4 (3.48)		
Oral hypoglycemic drugs combined with insulin therapy	148 (49.33)	89 (48.11)	59 (51.30)		
Oral hypoglycemic drugs	105 (35.00)	73 (39.46)	32 (27.83)		
Insulin therapy	39 (13.00)	19 (10.27)	20 (17.39)		
FPG (mmol/L), M (Q_1_, Q_3_)	7.09 (5.97, 9.16)	7.13 (6.09, 9.63)	6.99 (5.75, 8.58)	Z = -1.613	0.107
2hPG (mmol/L), Mean ± SD	16.91 ± 4.96	17.49 ± 5.00	15.97 ± 4.78	t = 2.60	0.010
HbAlc (%), Mean ± SD	7.92 ± 1.70	7.97 ± 1.68	7.84 ± 1.73	t = 0.67	0.504
TC (mmol/L), Mean ± SD	4.03 ± 1.01	4.11 ± 0.99	3.90 ± 1.05	t = 1.77	0.077
TG (mmol/L), M (Q_1_, Q_3_)	1.30 (1.00, 1.79)	1.30 (1.02, 1.81)	1.27 (0.88, 1.77)	Z = -1.229	0.219
LDL (mmol/L), M (Q_1_, Q_3_)	2.25 (1.76, 2.93)	2.31 (1.82, 3.05)	2.12 (1.52, 2.78)	Z = -2.246	0.025
HDL (mmol/L), Mean ± SD	1.11 ± 0.31	1.09 ± 0.29	1.14 ± 0.34	t = -1.24	0.216
QOL, Mean ± SD	54.43 ± 7.12	56.78 ± 6.18	50.65 ± 6.91	t = 7.98	< 0.001
MMAS-8, Mean ± SD	6.08 ± 1.80	6.30 ± 1.77	5.72 ± 1.80	t = 2.74	0.007

*BMI* Body mass index, *T2DM* Type 2 diabetes mellitus, *FPG* Fasting plasma glucose, *2hPG* 2-Hour postprandial glucose, *HbA1c* Glycated hemoglobin, *TC* Total cholesterol, *TG* Triglycerides, *LDL* Low density lipoprotein, *HDL* High density lipoprotein, *QOL* Quality of life, *MMAS* MORISKY Medication adherence scale

### Associations of depressive symptoms with medication adherence in older adults with T2DM

The association between depressive symptoms and medication adherence was first analyzed through evaluating the associations between depressive symptoms and the overall MMAS-8 as well as 8 items of MMAS-8. As exhibited in Table 2, after adjusting for age and gender, patients with depressive symptoms might be associated with decreased medication adherence compared with those without depressive symptoms (β = -0.64, 95%CI: -1.04, -0.23). Decreased medication adherence was observed in those with depressive symptoms (β = -0.67, 95%CI: -1.10, -0.24) after adjusting for age, gender, BMI, LDL and 2hPG. Depressive symptoms showed significant associations with decreased MMAS-8 in item 1 (β = -0.20, 95%CI: -0.32, -0.09), item 2 (β = -0.12, 95%CI: -0.22, -0.02) and item 4 (β = -0.12, 95%CI: -0.23, -0.01) after adjusting for age, gender, BMI, LDL and 2hPG (Table 2).

**Table 2:** Associations of depressive symptoms with medication compliance or QOL in older adults with T2DM

Outcomes	Model 1		Model 2		Model 3	
	β (95%CI)	*P*	β (95%CI)	*P*	β (95%CI)	*P*
MMAS-8	-0.58 (-1.00, -0.16)	0.007	-0.64 (-1.04, -0.23)	0.002	-0.67 (-1.10,-0.24)	0.002
M1	-0.17 (-0.28, -0.05)	0.004	-0.18 (-0.30, -0.07)	0.002	-0.20 (-0.32, -0.09)	< 0.001
M2	-0.11 (-0.20, -0.01)	0.025	-0.11 (-0.21, -0.02)	0.018	-0.12 (-0.22, -0.02)	0.015
M3	-0.07 (-0.17, 0.03)	0.185	-0.07 (-0.18, 0.03)	0.158	-0.08 (-0.18, 0.03)	0.150
M4	-0.10 (-0.21, 0.01)	0.060	-0.12 (-0.22, -0.01)	0.037	-0.12 (-0.23, -0.01)	0.038
M5	-0.02 (-0.10, 0.07)	0.654	-0.02 (-0.11, 0.06)	0.615	-0.01 (-0.09, 0.09)	0.971
M6	-0.01 (-0.09, 0.07)	0.804	-0.01 (-0.10, 0.07)	0.739	-0.01 (-0.10, 0.08)	0.817
M7	-0.06 (-0.16, 0.04)	0.246	-0.08 (-0.18, 0.03)	0.146	-0.09 (-0.19, 0.02)	0.106
M8	-0.04 (-0.08, 0.01)	0.116	-0.04 (-0.09, 0.01)	0.087	-0.05 (-0.10, 0.01)	0.051
QOL	-6.13 (-7.64, -4.62)	< 0.001	-6.11 (-7.63, -4.60)	< 0.001	-5.99 (-7.56, -4.42)	< 0.001
Q1	-0.41 (-0.60, -0.21)	< 0.001	-0.41 (-0.61, -0.22)	< 0.001	-0.36 (-0.57, -0.16)	< 0.001
Q2	-0.36 (-0.59, -0.13)	0.002	-0.37 (-0.60, -0.14)	0.002	-0.37 (-0.61, -0.13)	0.003
Q3	-2.19 (-2.71, -1.67)	< 0.001	-2.15 (-2.66, -1.64)	< 0.001	-2.09 (-2.62, -1.56)	< 0.001
Q4	-1.70 (-2.18, -1.22)	< 0.001	-1.67 (-2.15, -1.19)	< 0.001	-1.64 (-2.13, -1.14)	< 0.001
Q5	-1.18 (-1.65, -0.70)	< 0.001	-1.22 (-1.69, -0.74)	< 0.001	-1.25 (-1.74, -0.76)	< 0.001
Q6	-0.29 (-0.40, -0.18)	< 0.001	-0.29 (-0.41, -0.18)	< 0.001	-0.28 (-0.40, -0.16)	< 0.001

Model 1: Unadjusted univariate linear regression model

Model 2: Multivariable linear regression adjusted for age and gender

Model 3: Multivariable linear regression adjusted for age, gender, BMI, LDL and 2hPG

*QOL* Quality of life, *T2DM* Type 2 diabetes mellitus, *MMAS* Morisky medication adherence scale

### Associations of depressive symptoms with QOL in older adults with T2DM

In the crude model, depressive symptoms might correlate with reduced QOL in older adults with T2DM (β = -6.13, 95%CI: -7.64, -4.62). After adjusting for age and gender, depressive symptoms might be linked with QOL in older adults with T2DM (β = -6.11, 95%CI: -7.63, -4.60). After the adjustment for age, gender, BMI, LDL and 2hPG, depressive symptoms were associated with decreased QOL in older adults with T2DM (β = -5.99, 95%CI: -7.56, -4.42). We also found the association between depressive symptoms and decreased QOL in six items used in our study (Table 2).

### Interaction effect between medication adherence and depressive symptoms on QOL in older adults with T2DM

As exhibited in Table 3, compared with patient with MMAS-8 < 6 and depression, those with MMAS-8 ≥ 6 and depression (β = 3.95, 95%CI: 1.42, 6.48), MMAS-8 < 6 and no depression (β = 6.89, 95%CI: 4.21, 9.57), MMAS-8 ≥ 6 and no depression (β = 8.57, 95%CI: 6.24, 10.91) were associated with higher QOL in older adults with T2DM. The results of multiplicative interaction analysis revealed that there was no interaction effect between medication adherence and depressive symptoms on the QOL in older adults with T2DM (*P* > 0.05).

**Table 3:** Interaction effect between medication adherence and depressive symptoms on QOL in older adults with T2DM

Variables	Model 1		Model 2		Model 3	
	β (95%CI)	*P*	β (95%CI)	*P*	β (95%CI)	*P*
MMAS-8*Depression						
Level 1	Reference					
Level 2	3.43 (0.95, 5.92)	0.007	3.58 (1.07, 6.09)	0.005	3.95 (1.42, 6.48)	0.002
Level 3	6.98 (4.34, 9.61)	< 0.001	6.86 (4.22, 9.50)	< 0.001	6.89 (4.21, 9.57)	< 0.001
Level 4	8.36 (6.09, 10.63)	< 0.001	8.47 (6.20, 10.73)	< 0.001	8.57 (6.24, 10.91)	< 0.001
MMAS-8*Depression						
MMAS-8	1.39 (-0.62, 3.78)	0.175	1.60 (-0.46, 3.67)	0.127	1.68 (-0.30, 3.66)	0.095
Depression	-9.03 (-14.62, -3.44)	0.002	-8.84 (-14.43, -3.25)	0.002	-9.15 (-14.77, -3.54)	0.002
MMAS-8*Depression	2.05 (-1.15, 5.25)	0.208	1.98 (-1.22, 5.17)	0.224	2.26 (-0.93, 5.46)	0.165

Level 1: MMAS-8 < 6 & Depression, Level 2: MMAS-8 ≥ 6 & Depression, Level 3: MMAS-8 < 6 & Non-depression, and Level 4: MMAS-8 ≥ 6 & Non-depression

Model 1: Unadjusted univariate linear regression model

Model 2: Multivariable linear regression adjusted for age and gender

Model 3: Multivariable linear regression adjusted for age, gender, BMI, LDL and 2hPG

*QOL* Quality of life, *T2DM* Type 2 diabetes mellitus, *MMAS* Morisky medication adherence scale

### Mediating effect of medication adherence on depressive symptoms and QOL

**Fig. 1:**
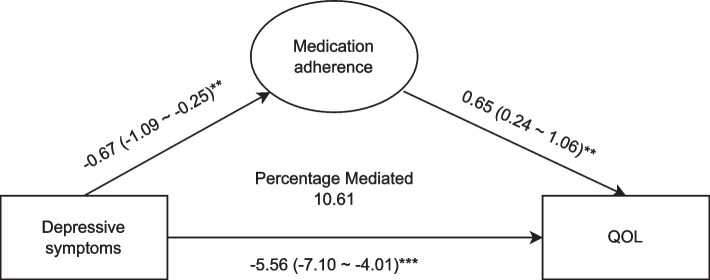
The mediating effect of medication adherence on depressive symptoms and QOL in older adults with T2DM

The results of mediating effect analysis indicated that depressive symptoms were associated with decreased medication adherence (β = -0.67, 95%CI: -1.09, -0.25). Medication adherence was linked with increased QOL of older adults with T2DM (β = 0.65, 95%CI: 0.24, 1.06). Depressive symptoms were correlated with decreased QOL of older adults with T2DM (β = -5.56, 95%CI: -7.10, -4.01). The percentage mediated by medication adherence on depressive symptoms and QOL in older adults with T2DM was 10.61% (Fig. 1). As shown in Supplementary Table 6, the mediating effect was further analyzed in the subgroup items with statistical difference. The results revealed that the percentage mediated by M1 on depressive symptoms and Q3 (Supplementary Fig. 1A), Q5 (Supplementary Fig. 1B), Q6 (Supplementary Fig. 1C) in older adults with T2DM was 6.55%, 15.54% and 15.57%, respectively. The percentage mediated by M4 on depressive symptoms and Q6 in older adults with T2DM was 7.12% (Supplementary Fig. 1D).

## Discussion

This cross-sectional study included 300 old adults with T2DM to explore the role of medication adherence in the association between depressive symptoms and QOL in older adults with T2DM. The data depicted that depressive symptoms were associated with decreased medication adherence and QOL in older adults with T2DM. Medication adherence played a mediating role on depressive symptoms and QOL in older adults with T2DM. The findings of the present study might provide a reference for the clinicians to offer early interventions to improve QOL of older adults with T2DM.

In previous studies, depression was frequently identified in DM patients, as DM can cause multiplex psychosocial problems in patients and in turn inhibits patients to fulfill their daily responsibilities such as adequate glycemic control and adherence to treatments [27]. The prevalence of depression was increased in T2DM patients, which was further associated with blood glucose fluctuation [28]. In T2DM patients with depression, non-adherence to pharmacological treatment is high compared with those free from symptoms of depression or any other alteration of their emotional state [29]. There was evidence indicated that depression decreased the diabetes self-care, self-management, behaviors including medication self-administration [30]. A nested randomized controlled trial depicted that patients with diabetes mellitus complicated with depression was associated with poorer adherence to hypoglycaemic medication, resulting in poorer glycaemic control, diabetes management, and increased complications [31]. Lunghi et al. revealed that depression was associated with a 1.52-fold increase in the risk of non-persistence with antidiabetic drugs in new users of antidiabetic drugs with depression [32]. Depressed mood in T2DM patients inhibited adherence to self-care behaviors by decreasing the desire to seek treatment [33]. These findings supported the results in our study, which showed that depressive symptoms were associated with decreased medication adherence in older adults with T2DM.

Accinelli et al. evaluated the frequency of depression and QOL in patients with DM and found that QOL was lower in patients with DM and depression [34]. Qubekile et al. depicted that in DM patients with or without HIV infection, the QOL was influenced by moderate to severe depressive symptoms [35]. In the present study, we found that depressive symptoms were associated with lower QOL in older adults with T2DM. This because patients may be fear of the disease and treatment-related complications such as diabetes micro- or macrovascular complications and hypoglycemia that negatively affect the QOL of patients [36]. T2DM and its complications have an economic influence on patients and their families through direct medical costs and loss of work and wages, and the treatment cost of patients with DM and depression was higher than the treatment of DM alone [37], which might decrease the QOL of patients.

In this study, we first evaluated the interaction effect between medication adherence and depressive symptoms on the QOL in older adults with T2DM, and found that there was no interaction effect between medication adherence and depressive symptoms on the QOL in older adults with T2DM. Furthermore, medication adherence was identified to have a mediating effect on depressive symptoms and QOL in older adults with T2DM. Previous studies revealed that HIV patients with depression had poor antiretroviral therapy adherence and low health-related QOL [38]. Pouwer et al. indicated that DM patients who were complicated with depression were more likely to have poor glycemic control [39], which might increase the risk of complications and result in poor QOL. Multiply studies revealed that the treatment adherence associated with QOL in DM patients [40, 41]. The findings in our study suggested that to early identify mental health problems in older people with T2DM is important as they may interfere substantially with the treatment and management of T2DM, and the complications of T2DM such as nephropathy or retinopathy [42]. Medication adherence is essential for the treatment of T2DM patients especially old adults, as they were vulnerable with high risk of depression which might lead to higher non-adherence [43]. Our study identified the mediating effect of medication adherence on depressive symptoms and QOL of T2DM patients, which indicated that clinicians should pay attention on mental health of T2DM patients, and early screening those with depressive symptoms in older adult patients with T2DM might provide potential improvements of the medication adherence and also might help improve the QOL of these patients [44].

In our study, the percentage mediated by medication adherence on depressive symptoms and QOL in older adults with T2DM was 10.61%, and depressive symptoms had a strong direct association with the QOL in older adults with T2DM. This indicated that there is a need to plan awareness and counseling programs followed by regular follow-up to identify those with high risk of depressive symptoms, and for older adults with T2DM who were already diagnosed with depressive symptoms, anti-depressive drugs should be timely used if necessary. Medication adherence mediated the association between depressive symptoms and the QOL in older adults with T2DM, which indicated that medication adherence was also important for the QOL in older adults with T2DM, and clinicians should develop strategies to facilitate medication taking, and to provide ongoing support and assessment of adherence at each visit. Additionally, although no significant interaction effect between medication adherence and depressive symptoms on the QOL in older adults with T2DM, those with no depressive symptoms and high medication adherence was associated with better QOL in older adults with T2DM compared with those who had depressive symptoms and low medication adherence. These findings suggested early prevention should be provided for depressive symptoms and low medication adherence in older adults with T2DM, and timely identification for those with depressive symptoms and low medication adherence is necessary so that interventions for anti-depression and improving medication adherence could be provided.

Subgroup analysis on mediating effect of medication adherence on depressive symptoms and QOL in older adults with T2DM depicted that M1 (Do you sometimes forget to take your hypoglycemic medicine), M4 (When you travel or leave home, do you sometimes forget to bring along your medications) might play significant part on mediating the depressive symptoms and physical, social, and environmental domains of QOL in older adults with T2DM. The results might more clearly identify which items had mediating effects on the specific domains of QOL and provide a reference for making detailed plans to improve the QOL of older adults with T2DM.

There were several limitations in the current study. Firstly, this was a cross-sectional study, we could only identify the associations between depressive symptoms and medication adherence or QOL in older adult patients with T2DM, and causality between depressive symptoms and medication adherence or QOL in older adult patients with T2DM could not be found. Although the directions of the causal paths between the variables were unclear, based on results from previous cross-sectional studies, depression was identified to be associated with QOL of patients with dialysis [45]. Depression had effects on adherence to treatment and QOL in patients with end-stage renal disease undergoing dialysis, and adherence to treatment was also associated with QOL in these patients [46]. Thus we suspected that there might be a mediating effect of medication adherence on depressive symptoms and QOL of older adults with T2DM. Secondly, the sample size was small, and in the future, studies with more sample size were required to validate the results in the present study.

## Conclusions

The current study evaluated the mediating effect of medication adherence on depressive symptoms and QOL of older adults with T2DM and found medication adherence might mediate depressive symptoms and QOL of older adults with T2DM. The findings suggested that the clinicians should concern on the mental status of older adults with T2DM, and early identified those with high risk of depressive symptoms and provide timely interventions.

## Supplementary Information

**Additional file 1: Supplementary Table 1**. The detailed information of MMAS-8. **Supplementary Table 2**. The detailed information of WHOQOL-BREF. **Supplementary Table 3**. The internal consistency of MMAS-8 and WHOQOL-BREF. **Supplementary Table 4**. Sensitivity analysis of variables before and after the missing values manipulated. **Supplementary Table 5**. Comparisons of characteristics of T2DM patients with MMAS-8<6 or ≥6. **Supplementary Table 6.** Subgroup analysis on the mediating effect of mediation adherence on symptoms and QOL of older adults with T2DM.

**Additional file 2:** **Supplementary Figure 1.** Subgroup analysis on the mediating effect of mediation adherence on symptoms and QOL of older adults with T2DM.

## Data Availability

The datasets used and/or analyzed during the current study are available from the corresponding author on reasonable request.

## Abbreviations

DM: Diabetes mellitus
T2DM: Type 2 diabetes mellitus
QOL: Quality of life
COPD: Chronic obstructive pulmonary disease
WHO: World Health Organization
BMI: Body mass index
FPG: Fasting plasma glucose
2Hpg: 2-Hour postprandial glucose
TC: Total cholesterol
TG: Triglycerides
LDL: Low density lipoprotein
HDL: High density lipoprotein
HAMD-17: Hamilton Depression Scale-17
MMAS-8: Morisky Medication Adherence Scale-8
WHOQOL-BREF: World Health Organization Quality of Life questionnaire
SD: Standard deviation
CI: Confidence interval
