# Supplementary material for: The role of medication adherence in the association between depressive symptoms and quality of life in older adults with type 2 diabetes mellitus
Source: BMC Geriatr. 2023 Mar 30;23:196. doi: 10.1186/s12877-023-03929-8 (PMC10064516; doi:10.1186/s12877-023-03929-8)
Supplement: Supplementary file 1 — Additional file 1: Supplementary Table 1. The detailed information of MMAS-8. Supplementary Table 2. The detailed information of WHOQOL-BREF. Supplementary Table 3. The internal consistency of MMAS-8 and WHOQOL-BREF. Supplementary Table 4. Sensitivity analysis of variables before and after the missing values manipulated. Supplementary Table 5. Comparisons of characteristics of T2DM patients with MMAS-8<6 or ≥6. Supplementary Table 6. Subgroup analysis on the mediating effect of mediation adherence on symptoms and QOL of older adults with T2DM. [file 12877_2023_3929_MOESM1_ESM.docx]

Supplementary Table 1 The detailed information of MMAS-8

| Questions | Points-Answers |
| --- | --- |
| 1. Do you sometimes forget to take your hypoglycemic medicine？ | 0 score——Yes  1 score——No |
| 2. Over the past 2 weeks, were there any days when you did not take your hypoglycemic medicine？ | 0 score——Yes  1 score——No |
| 3. Have you ever cut back or stopped taking your medication without telling your doctor because you felt worse when you took it？ | 0 score——Yes  1 score——No |
| 4. When you travel or leave home, do you sometimes forget to bring along your medications？ | 0 score——Yes  1 score——No |
| 5. Did you take your hypoglycemic medicine yesterday？ | 0 score——Yes  1 score——No |
| 6. When you feel like your blood sugar is under control, do you sometimes stop taking your medicine？ | 0 score——Yes  1 score——No |
| 7. Taking medication everyday is a real inconvenience for some people. Do you ever feel hassled about sticking to your diabetes treatment plan？ | 0 score——Yes  1 score——No |
| 8. How often do you have difficulty remembering to take all your hypoglycemic medicine？ | 1 score——Never  0.75 score——Once in while  0.5 score——Sometimes  0.25 score——Usually  0 score——All the time |

Supplementary Table 2 The detailed information of WHOQOL-BREF

| We ask that you think about your life in the last four weeks. | | | | | | |
| --- | --- | --- | --- | --- | --- | --- |
|  |  | Very poor | Poor | Neither poor nor good | Good | Very good |
| 1. | How would you rate your quality of life? | 1 | 2 | 3 | 4 | 5 |
|  |  | Very dissatisfied | Dissatisfied | Neither satisfied nor dissatisfied | Satisfied | Very satisfied |
| 2. | How satisfied are you with your health? | 1 | 2 | 3 | 4 | 5 |
| The following questions ask about how much you have experienced certain things in the last four weeks. | | | | | | |
|  |  | Not at all | A little | A moderate amount | Very much | An extreme amount |
| 3. | To what extent do you feel that physical pain prevents you from  doing what you need to do? | 5 | 4 | 3 | 2 | 1 |
| 4. | How much do you need any medical treatment to function in your daily life? | 5 | 4 | 3 | 2 | 1 |
| 5. | How much do you enjoy life? | 1 | 2 | 3 | 4 | 5 |
| 6. | To what extent do you feel your life to be meaningful? | 1 | 2 | 3 | 4 | 5 |
|  |  | Not at all | A little | A moderate amount | Very much | Extremely |
| 7. | How well are you able to concentrate? | 1 | 2 | 3 | 4 | 5 |
| 8. | How safe do you feel in your daily life? | 1 | 2 | 3 | 4 | 5 |
| 9. | How healthy is your physical environment? | 1 | 2 | 3 | 4 | 5 |
| The following questions ask about how completely you experience or were able to do certain things in the last four weeks. | | | | | | |
|  |  | Not at all | A little | Moderately | Mostly | Completely |
| 10. | Do you have enough energy for everyday life? | 1 | 2 | 3 | 4 | 5 |
| 11. | Are you able to accept your bodily appearance? | 1 | 2 | 3 | 4 | 5 |
| 12. | Have you enough money to meet your needs? | 1 | 2 | 3 | 4 | 5 |
| 13. | How available to you is the information that you need in your day-to-day life? | 1 | 2 | 3 | 4 | 5 |
| 14. | To what extent do you have the opportunity for leisure activities? | 1 | 2 | 3 | 4 | 5 |
|  |  | Very poor | Poor | Neither poor nor good | Good | Very good |
| 15. | How well are you able to get around? | 1 | 2 | 3 | 4 | 5 |
|  |  | Very dissatisfied | Dissatisfied | Neither satisfied nor dissatisfied | Satisfied | Very satisfied |
| 16. | How satisfied are you with your sleep? | 1 | 2 | 3 | 4 | 5 |
| 17. | How satisfied are you with your ability to perform your daily living activities? | 1 | 2 | 3 | 4 | 5 |
| 18. | How satisfied are you with your capacity for work? | 1 | 2 | 3 | 4 | 5 |
| 19. | How satisfied are you with yourself? | 1 | 2 | 3 | 4 | 5 |
| 20. | How satisfied are you with your personal relationships? | 1 | 2 | 3 | 4 | 5 |
| 21. | How satisfied are you with your sex life? | 1 | 2 | 3 | 4 | 5 |
| 22. | How satisfied are you with the support you get from your friends? | 1 | 2 | 3 | 4 | 5 |
| 23. | How satisfied are you with the conditions of your living place? | 1 | 2 | 3 | 4 | 5 |
| 24. | How satisfied are you with your access to health services? | 1 | 2 | 3 | 4 | 5 |
| 25. | How satisfied are you with your transport? | 1 | 2 | 3 | 4 | 5 |
| The following question refers to how often you have felt or experienced certain things in the last four weeks. | | | | | | |
|  |  | Never | Seldom | Quite often | Very often | Always |
| 26. | How often do you have negative feelings such as blue mood, despair, anxiety, depression? | 5 | 4 | 3 | 2 | 1 |

Supplementary Table 3 The internal consistency of MMAS-8 and WHOQOL-BREF

| **Scales** | **R** | **Alpha** |  |
| --- | --- | --- | --- |
| **MMAS** |  | **0.68** |  |
| M_1_ | 0.51 | 0.61 |  |
| M_2_ | 0.48 | 0.62 |  |
| M_3_ | 0.41 | 0.64 |  |
| M_4_ | 0.43 | 0.63 |  |
| M_5_ | 0.38 | 0.61 |  |
| M_6_ | 0.44 | 0.64 |  |
| M_7_ | 0.26 | 0.68 |  |
| M_8_ | 0.43 | 0.66 |  |
| **QOL** |  | **0.79** |  |
| Q_1_ | 0.62 | 0.76 |  |
| Q_2_ | 0.48 | 0.78 |  |
| Q_3_ | 0.64 | 0.74 |  |
| Q_4_ | 0.76 | 0.69 |  |
| Q_5_ | 0.57 | 0.75 |  |
| Q_6_ | 0.74 | 0.78 |  |

Supplementary Table 4 Sensitivity analysis of variables before and after the missing values manipulated

| Variables | Missing values, n (%) | After imputation | Before imputation | Statistics | *P* |
| --- | --- | --- | --- | --- | --- |
| Gender | 0 (0.00) |  |  |  |  |
| Age | 0 (0.00) |  |  |  |  |
| Height | 0 (0.00) |  |  |  |  |
| Weight | 0 (0.00) |  |  |  |  |
| BMI | 0 (0.00) |  |  |  |  |
| Marital status | 0 (0. 00) |  |  |  |  |
| Education | 0 (0.00) |  |  |  |  |
| Living status | 0 (0.00) |  |  |  |  |
| Smoking | 0 (0.00) |  |  |  |  |
| Drinking | 0 (0.00) |  |  |  |  |
| Duration of T2DM | 0 (0.00) |  |  |  |  |
| Complicated with hypertension | 0 (0.00) |  |  |  |  |
| Complicated with dyslipidemia | 0 (0.00) |  |  |  |  |
| Complicated with other diseases | 0 (0.00) |  |  |  |  |
| First-degree family history of diabetes | 0 (0.00) |  |  |  |  |
| Diabetic retinopathy | 0 (0.00) |  |  |  |  |
| Diabetic peripheral neuropathy | 0 (0.00) |  |  |  |  |
| Diabetic peripheral vasculopathy | 0 (0.00) |  |  |  |  |
| Diabetic ketoacidosis | 0 (0.00) |  |  |  |  |
| Diabetic nephropathy | 0 (0.00) |  |  |  |  |
| Treatments for T2DM | 0 (0.00) |  |  |  |  |
| FPG | 0 (0.00) |  |  |  |  |
| 2hPG | 0 (0.00) |  |  |  |  |
| HbAlc | 0 (0.00) |  |  |  |  |
| TC | 2 (0.67) | 4.03±1.01 | 4.03±1.01 | t=-0.02 | 0.983 |
| TG | 2 (0.67) | 1.30 (1.00,1.79) | 1.30 (1.00,1.79) | Z=0.093 | 0.926 |
| LDL-C | 2 (0.67) | 2.25 (1.76,2.93) | 2.25 (1.75,2.91) | Z=-0.014 | 0.989 |
| HDL-C | 2 (0.67) | 1.11±0.31 | 1.11±0.31 | t=-0.02 | 0.987 |
| QOL | 0 (0.00) |  |  |  |  |
| MMAS-8 | 0 (0.00) |  |  |  |  |
| Depression | 0 (0.00) |  |  |  |  |

BMI: body mass index, T2DM: type 2 diabetes mellitus, FPG: fasting plasma glucose, HbA1c: glycated hemoglobin, TC: total cholesterol, TG: triglycerides, LDL: low density lipoprotein, HDL: high density lipoprotein, QOL: quality of life, MMAS-8: Morisky Medication Adherence Scale-8

Supplementary Table 5 Comparisons of characteristics of T2DM patients with MMAS-8<6 or ≥6

| Variables | Total (n=300) | MMAS-8<6 (n=114) | MMAS-8≥6 (n=186) | Statistics | *P* |
| --- | --- | --- | --- | --- | --- |
| Gender, n (%) |  |  |  | χ^2^=6.396 | 0.011 |
| Male | 186 (62.00) | 81 (71.05) | 105 (56.45) |  |  |
| Female | 114 (38.00) | 33 (28.95) | 81 (43.55) |  |  |
| Age (years), Mean ± SD | 70.60 ± 8.25 | 69.39 ± 8.00 | 71.35 ± 8.34 | t=-2.01 | 0.045 |
| Height (m), Mean ± SD | 1.66 ± 0.08 | 1.68 ± 0.08 | 1.65 ± 0.08 | t=2.59 | 0.010 |
| Weight (kg), Mean ± SD | 67.19 ± 10.57 | 68.54 ± 10.32 | 66.36 ± 10.67 | t=1.74 | 0.083 |
| BMI (kg/m^2^), Mean ± SD | 24.32 ± 3.05 | 24.38 ± 3.06 | 24.29 ± 3.05 | t=0.25 | 0.802 |
| Marital status, n (%) |  |  |  | - | 0.008 |
| Divorced | 5 (1.67) | 4 (3.51) | 1 (0.54) |  |  |
| Widowed | 32 (10.67) | 6 (5.26) | 26 (13.98) |  |  |
| Married | 263 (87.67) | 104 (91.23) | 159 (85.48) |  |  |
| Education, n (%) |  |  |  | χ^2^=1.429 | 0.232 |
| High school or above | 198 (66.00) | 80 (70.18) | 118 (63.44) |  |  |
| Under the high school | 102 (34.00) | 34 (29.82) | 68 (36.56) |  |  |
| Living alone, n (%) |  |  |  | χ^2^=0.632 | 0.427 |
| Yes | 26 (8.67) | 8 (7.02) | 18 (9.68) |  |  |
| No | 274 (91.33) | 106 (92.98) | 168 (90.32) |  |  |
| Smoking, n (%) |  |  |  | χ^2^=2.474 | 0.290 |
| Never | 180 (60.00) | 62 (54.39) | 118 (63.44) |  |  |
| Smoking at present | 47 (15.67) | 21 (18.42) | 26 (13.98) |  |  |
| Smoking before | 73 (24.33) | 31 (27.19) | 42 (22.58) |  |  |
| Drinking, n (%) |  |  |  | χ^2^=0.521 | 0.771 |
| Never | 162 (54.00) | 59 (51.75) | 103 (55.38) |  |  |
| Drinking at present | 60 (20.00) | 25 (21.93) | 35 (18.82) |  |  |
| Drinking before | 78 (26.00) | 30 (26.32) | 48 (25.81) |  |  |
| Duration of T2DM (year), M (Q_1_, Q_3_) | 15.00 (8.50, 20.00) | 15.00 (8.00, 20.00) | 16.00 (9.00, 20.00) | Z=-1.617 | 0.106 |
| Hypertension, n (%) |  |  |  | χ^2^=0.201 | 0.654 |
| No | 85 (28.33) | 34 (29.82) | 51 (27.42) |  |  |
| Yes | 215 (71.67) | 80 (70.18) | 135 (72.58) |  |  |
| Dyslipidemia, n (%) |  |  |  | χ^2^=1.761 | 0.185 |
| No | 125 (41.67) | 42 (36.84) | 83 (44.62) |  |  |
| Yes | 175 (58.33) | 72 (63.16) | 103 (55.38) |  |  |
| Other diseases, n (%) |  |  |  | χ^2^=0.059 | 0.809 |
| No | 44 (14.67) | 16 (14.04) | 28 (15.05) |  |  |
| Yes | 256 (85.33) | 98 (85.96) | 158 (84.95) |  |  |
| First-degree family history of diabetes, n (%) |  |  |  | χ^2^=0.713 | 0.398 |
| No | 175 (58.33) | 70 (61.40) | 105 (56.45) |  |  |
| Yes | 125 (41.67) | 44 (38.60) | 81 (43.55) |  |  |
| Diabetic retinopathy, n (%) |  |  |  | χ^2^=0.012 | 0.914 |
| No | 273 (91.00) | 104 (91.23) | 169 (90.86) |  |  |
| Yes | 27 (9.00) | 10 (8.77) | 17 (9.14) |  |  |
| Diabetic peripheral neuropathy, n (%) |  |  |  | χ^2^=6.190 | 0.013 |
| No | 108 (36.00) | 31 (27.19) | 77 (41.40) |  |  |
| Yes | 192 (64.00) | 83 (72.81) | 109 (58.60) |  |  |
| Diabetic peripheral vasculopathy, n (%) |  |  |  | - | 0.291 |
| No | 3 (1.00) | 0 (0.00) | 3 (1.61) |  |  |
| Yes | 297 (99.00) | 114 (100.00) | 183 (98.39) |  |  |
| Diabetic ketoacidosis, n (%) |  |  |  | - | 1.000 |
| No | 298 (99.33) | 113 (99.12) | 185 (99.46) |  |  |
| Yes | 2 (0.67) | 1 (0.88) | 1 (0.54) |  |  |
| Diabetic nephropathy, n (%) |  |  |  | χ^2^=0.015 | 0.904 |
| No | 175 (58.33) | 67 (58.77) | 108 (58.06) |  |  |
| Yes | 125 (41.67) | 47 (41.23) | 78 (41.94) |  |  |
| Treatments for T2DM, n (%) |  |  |  | - | 0.040 |
| Non-drug therapy | 8 (2.67) | 7 (6.14) | 1 (0.54) |  |  |
| Oral hypoglycemic drugs combined with insulin therapy | 148 (49.33) | 53 (46.49) | 95 (51.08) |  |  |
| Oral hypoglycemic drugs | 105 (35.00) | 39 (34.21) | 66 (35.48) |  |  |
| Insulin therapy | 39 (13.00) | 15 (13.16) | 24 (12.90) |  |  |
| FPG (mmol/L), M (Q_1_, Q_3_) | 7.09 (5.97, 9.16) | 7.09 (6.11, 8.99) | 7.07 (5.95, 9.25) | Z=0.099 | 0.921 |
| 2hPG (mmol/L), Mean ± SD | 16.91 ± 4.96 | 16.21 ± 4.97 | 17.34 ± 4.92 | t=-1.91 | 0.057 |
| HbAlc (%), Mean ± SD | 7.92 ± 1.70 | 7.87 ± 1.69 | 7.96 ± 1.71 | t=-0.44 | 0.663 |
| TC (mmol/L), Mean ± SD | 4.03 ± 1.01 | 4.09 ± 1.02 | 4.00 ± 1.01 | t=0.74 | 0.458 |
| TG (mmol/L), M (Q_1_, Q_3_) | 1.30 (1.00, 1.79) | 1.37 (1.04, 2.03) | 1.27 (0.97, 1.65) | Z=1.907 | 0.057 |
| LDL (mmol/L), M (Q_1_, Q_3_) | 2.25 (1.76, 2.93) | 2.25 (1.78, 3.10) | 2.26 (1.73, 2.85) | Z=0.365 | 0.715 |
| HDL (mmol/L), Mean ± SD | 1.11 ± 0.31 | 1.08 ± 0.28 | 1.13 ± 0.33 | t=-1.35 | 0.177 |
| Depressive symptoms |  |  |  | χ^2^=17.78 | <0.001 |
| Yes | 115 (38.3) | 55(47.83) | 60 (52.17) |  |  |
| No | 185 (61.7) | 59 (31.89) | 126 (68.11) |  |  |
| QOL, Mean ± SD | 54.43 ± 7.12 | 52.47 ± 7.94 | 55.63 ± 6.29 | t=-3.61 | <0.001 |

BMI: body mass index, T2DM: type 2 diabetes mellitus, FPG: fasting plasma glucose, 2hPG: 2-hour postprandial glucose, HbA1c: glycated hemoglobin, TC: total cholesterol, TG: triglycerides, LDL: low density lipoprotein, HDL: high density lipoprotein, QOL: quality of life, MMAS: Morisky Medication Adherence Scale

Supplementary Table 6 Subgroup analysis on the mediating effect of mediation adherence on symptoms and QOL of older adults with T2DM

|  | X-M | X-Y | M-Y | Percent |
| --- | --- | --- | --- | --- |
| **M1** |  |  |  |  |
| Q1 | -0.20 (-0.32 ~ -0.09)^*^ | -0.33 (-0.54 ~ -0.12) ^*^ | 0.15 (-0.04 ~ 0.35) | 8.70 |
| Q2 | -0.20 (-0.32 ~ -0.09) ^*^ | -0.35 (-0.59 ~ -0.11) ^*^ | 0.11 (-0.12 ~ 0.35) | 6.25 |
| Q3 | -0.20 (-0.32 ~ -0.08) ^*^ | -1.95 (-2.48 ~ -1.43) ^*^ | 0.67 (0.17-1.18) ^*^ | 6.55 |
| Q4 | -0.20 (-0.32 ~ -0.09) ^*^ | -1.55 (-2.05 ~ -1.05) ^*^ | 0.44 (-0.04 ~ 0.92) | 5.43 |
| Q5 | -0.20 (-0.32 ~ -0.09) ^*^ | -1.06 (-1.54 ~ -0.57) ^*^ | 0.96 (0.49 ~ 1.42) ^*^ | 15.54 |
| Q6 | -0.20 (-0.32 ~ -0.09) ^*^ | -0.24 (-0.36 ~ -0.12) ^*^ | 0.22 (0.10 ~ 0.33) ^*^ | 15.57 |
| **M2** |  |  |  |  |
| Q1 | -0.12 (-0.22 ~ -0.03) ^*^ | -0.36 (-0.57 ~ -0.16) ^*^ | -0.01 (-0.25 ~ 0.23) | 0.22 |
| Q2 | -0.12 (-0.22 ~ -0.03) ^*^ | -0.37 (-0.61 ~ -0.13) ^*^ | -0.02 (-0.30 ~ 0.37) | 0.52 |
| Q3 | -0.12 (-0.22 ~ -0.03) ^*^ | -2.03 (-2.56 ~ -1.51) ^*^ | 0.46 (-0.15 ~ 1.08) | 2.67 |
| Q4 | -0.12 (-0.22 ~ -0.03) ^*^ | -1.61 (-2.11 ~ -1.12) ^*^ | 0.21 (-0.38 ~ 0.79) | 1.52 |
| Q5 | -0.12 (-0.22 ~ -0.03) ^*^ | -1.24 (-1.74 ~ -0.76) ^*^ | 0.03 (-0.55 ~ 0.61) | 0.30 |
| Q6 | -0.12 (-0.22 ~ -0.03) ^*^ | -0.28 (-0.40 ~ -0.16) ^*^ | 0.06 (-0.08 ~ 0.20) | 2.69 |
| **M4** |  |  |  |  |
| Q1 | -0.12 (-0.23 ~ -0.01) ^*^ | -0.35 (-0.55 ~ -0.14) ^*^ | 0.12 (-0.09 ~ 0.32) | 3.92 |
| Q2 | -0.12 (-0.23 ~ -0.01) ^*^ | -0.38 (-0.62 ~ -0.14) ^*^ | -0.06 (-0.30 ~ 0.18) | 1.86 |
| Q3 | -0.12 (-0.23 ~ -0.01) ^*^ | -2.04 (-2.56 ~ -1.52) ^*^ | 0.41 (-0.11 ~ 0.94) | 2.39 |
| Q4 | -0.12 (-0.23 ~ -0.01) ^*^ | -1.60 (-2.09 ~ -1.11) ^*^ | 0.30 (-0.19 ~ 0.80) | 2.22 |
| Q5 | -0.12 (-0.23 ~ -0.01) ^*^ | -1.19 (-1.68 ~ -0.71) ^*^ | 0.47 (-0.02 ~ 0.96) | 4.55 |
| Q6 | -0.12 (-0.23 ~ -0.01) ^*^ | -0.26 (-0.38 ~ -0.15) ^*^ | 0.17 (0.05 ~ 0.29) ^*^ | 7.12 |

T2DM: type 2 diabetes mellitus, QOL: quality of life

* indicated there was statistical association between the variables.
