# Supplementary material for: The role of medication adherence in the association between depressive symptoms and quality of life in older adults with type 2 diabetes mellitus
Source: BMC Geriatr. 2023 Mar 30;23:196. doi: 10.1186/s12877-023-03929-8 (PMC10064516; doi:10.1186/s12877-023-03929-8)

Supplementary Figure 1 Subgroup analysis on the mediating effect of mediation adherence on symptoms and QOL of older adults with T2DM.

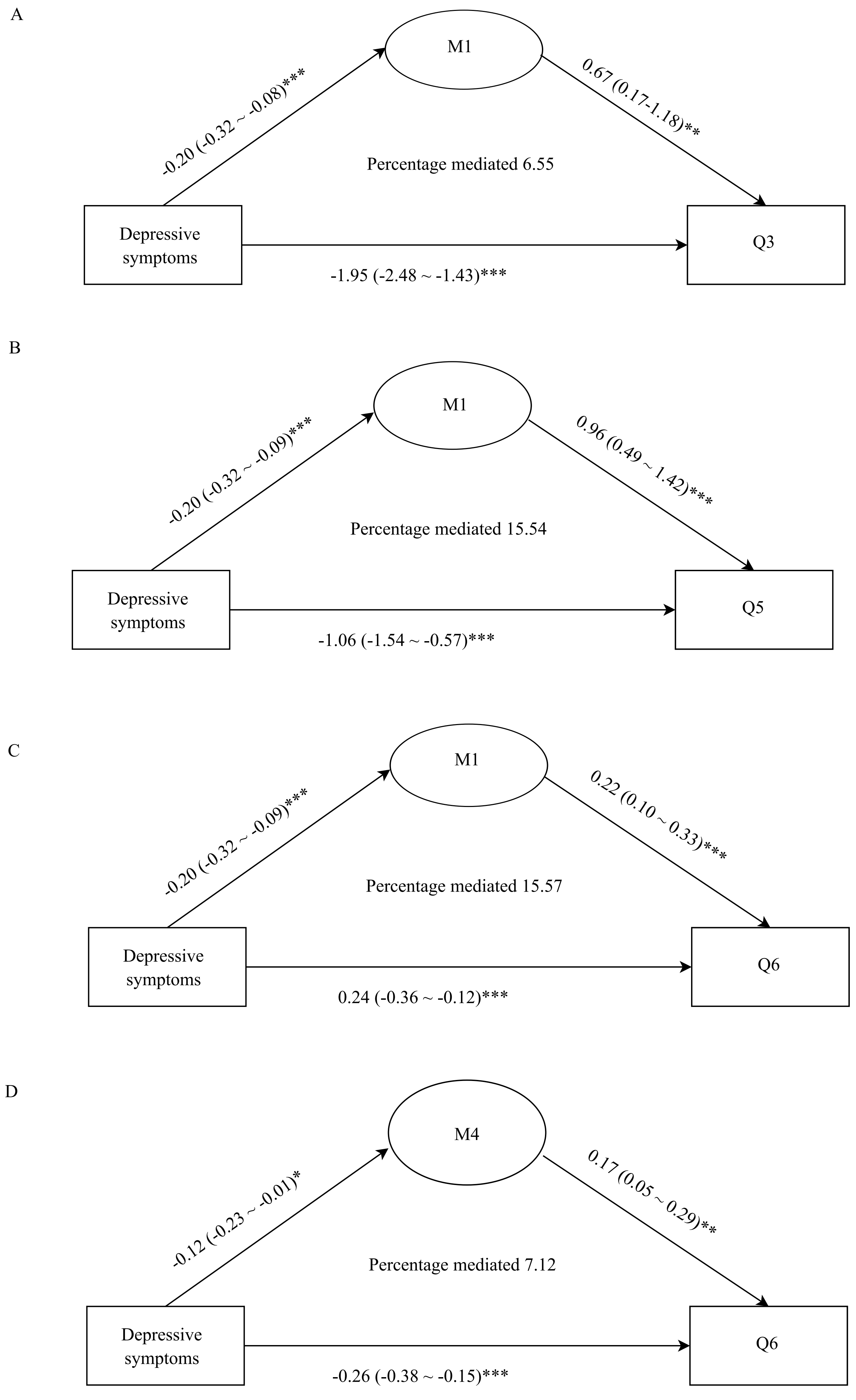

Supplement: Supplementary file 2 — Additional file 2: Supplementary Figure 1. Subgroup analysis on the mediating effect of mediation adherence on symptoms and QOL of older adults with T2DM. [file 12877_2023_3929_MOESM2_ESM.pdf]
